# Complete Genome Sequence of *Streptomyces* sp. Strain CA-256286

**DOI:** 10.1128/MRA.00290-21

**Published:** 2021-06-03

**Authors:** Charlotte Beck, Tetiana Gren, Tue S. Jørgensen, Ignacio González, Fernando Román-Hurtado, Daniel Oves-Costales, Olga Genilloud, Tilmann Weber

**Affiliations:** a The Novo Nordisk Foundation Center for Biosustainability, Technical University of Denmark, Kongens Lyngby, Denmark; b Fundación MEDINA, Granada, Spain; University of Maryland School of Medicine

## Abstract

Here, we report the sequencing, assembly, and annotation of the genome of *Streptomyces* sp. strain CA-256286. The genome consists of a linear 7,726,360-nucleotide chromosome and a linear 466,817-nucleotide putative plasmid. This strain is predicted to produce a range of novel secondary metabolites.

## ANNOUNCEMENT

*Streptomyces* sp. strain CA-256286 was isolated from a soil sample collected at Pilar de la Mola (Formentera, Spain) from a muddy torrent and was treated with dry heat (100°C for 1 h). The original colony was isolated from a soil serial dilution suspension plated onto M3 medium supplemented with nalidixic acid (20 mg/liter) (1), after incubation for 6 weeks at 28°C in 70% relative humidity. The strain was grown in yeast extract-malt extract (YEME) medium for DNA isolation according to the method described by Kieser et al. (2). The genomic DNA for sequencing was purified using the Genomic-tip 100 kit (Qiagen, Venlo, Netherlands). The genomic DNA was sheared using a g-TUBE (Covaris, Inc., Woburn, MA, USA), followed by BluePippin size selection (Sage Science, Beverly, MA, USA). Macrogen, Inc. (Seoul, South Korea), generated the RS II (Pacific Biosciences [PacBio], Menlo Park, CA, USA) data (87,704 subreads, with an *N*_50_ value of 18,359 nucleotides [nt]) using the single-molecule real-time (SMRT) Cell 8PAC v3, the DNA polymerase binding kit P6, and two SMRT cells. PacBio polymerase reads were partitioned to subreads by Macrogen using SMRT Analysis v2.3 (PacBio) (http://www.pacb.com/support/software-downloads), which removes adapter-related sequences. Default software parameters were used except where otherwise noted. Flye v2.8 (3) was used to assemble the PacBio subreads with the parameter --iterations 5 for a total of five rounds of polishing using the PacBio data, also including quality filtering, which excluded subreads shorter than 5,000 nt. The resulting assembly consists of two contigs. A 7,726,360-nt chromosome sequence (GenBank accession number CP071044) with inverted repeat chromosome ends of 26,587 nt, which is characteristic of *Streptomyces* strains, was identified. In addition, a 466,817-nt linear putative plasmid (GenBank accession number CP071045) was found. Both contigs were found to be linear, based on Bandage v0.8.1 (4) visualization of the Flye assembly graph. The coverages for the chromosome and the putative plasmid are 108× and 95×, respectively, indicating that the copy number for the putative plasmid is 1. The genome of *Streptomyces* sp. strain CA-256286 was annotated using Prokka v1.14.6 (5), with the switches --cdsrnaolap --rnammer (6) --increment 10. In addition to default databases, the Pfam-A v32.0 database was used along with the genomes of six actinobacterial species with manual or otherwise high-quality annotations (see reference 7 for details). The five genes closest to the chromosome ends all lack functional annotation. The chromosome harbors 6,744 protein-coding genes, 6 rRNA operons, and 79 tRNAs, and the putative plasmid contains 466 protein-coding genes. The GC content of the chromosome is 71.6%, and that of the plasmid is 68.4%. Our initial assessment of the production capabilities of *Streptomyces* sp. strain CA-256286 was supported by genome mining analyses with antiSMASH v6.0.0alpha1-60bffdb (8). In total, 41 regions were predicted, covering 6 polyketide synthase (PKS), 5 nonribosomal peptide synthetase (NRPS), 8 PKS/NRPS, 9 terpene, 1 melanin, 4 lassopeptide, 1 lanthipeptide, 2 butyrolactone, 2 siderophore, and 3 ectoine biosynthetic gene clusters. According to autoMLST (9), the closest relative is *Streptomyces* sp. NRRL S-623 (NCBI assembly accesion number GCF_000725705.1) with 99.4% estimated average nucleotide identity.

### Data availability.

Raw PacBio data and the assembly and annotation of the CA-256286 genome are available via BioProject PRJNA689435. The NCBI GenBank accession numbers are CP071044 (chromosome) and CP071045 (plasmid).
